# Pharmaceutical Prescribing Privileges for Optometrists to Combat Childhood Myopia in Singapore: Public Health Policy Review and Analysis

**DOI:** 10.3390/children11121548

**Published:** 2024-12-20

**Authors:** Tiong Peng Yap, Masuma Pervin Mishu

**Affiliations:** 1 IGARD Paediatric Optometry and Vision Therapy Centre, Singapore 229469, Singapore; 2 Institute of Epidemiology and Healthcare, Faculty of Population Health Sciences, University College London, London WC1E 6BT, UK

**Keywords:** myopia, evidence-based myopia interventions, public health policy review

## Abstract

Singapore’s national myopia prevention efforts have largely focused on school vision screening and public education on outdoor activities in the past two decades. Given the emergence of evidence-based myopia interventions, this policy review and analysis investigates the potential benefits and drawbacks of optometrist prescribing privileges as it has been proposed to reduce the barriers to access effective interventions, such as combined therapy (e.g., orthokeratology treatment and low-dose atropine therapy). In this policy analysis, two policy options were identified to be feasible based on evidence from a systematic literature search and they were analysed along with status quo using the Centers for Disease Control and Prevention (CDC) Policy Analysis Framework. This includes independent prescribing and supplementary prescribing, where the former entails autonomous clinical decision making, and the latter entails co-management with ophthalmological supervision. The policy review and analysis found independent prescribing the most favourable and concluded that this should be implemented in view of its benefits for the community. Public health impact is expected to be substantial due to increased patient access, reduced treatment costs, early interventions, improved treatment compliance, and reduced wait times and inconvenience. It is feasible because treatment processes can be streamlined, and it can be implemented based on existing collaborative prescribing frameworks. Economical and budgetary impact is also substantial given the direct savings generated, which can consequently help to reduce the disease burden.

## 1. Introduction

Myopia is a significant global public health issue according to the World Health Organization (WHO), owing to its widespread impact on visual loss, rapid progression, and risks of future eye diseases [1,2]. Its prevalence is particularly high in Singapore [3], as nearly 65% of school-aged children are myopic by the age of 12 years [2,4], and 13% of them have severely poor vision [4]. If this trend continues, it would affect 80% of adults [4], and 24% may suffer from high myopia [5], which predisposes them to complications such as macular degeneration [6], retinal detachment [7], glaucoma [8], and blindness [9]. Given the risks of disease complications, treatment costs of high myopia can be substantial to individuals and their families, also implicating economic and societal costs [10].

### 1.1. Current National Approach in Managing Childhood Myopia in Singapore

**Figure 1:**
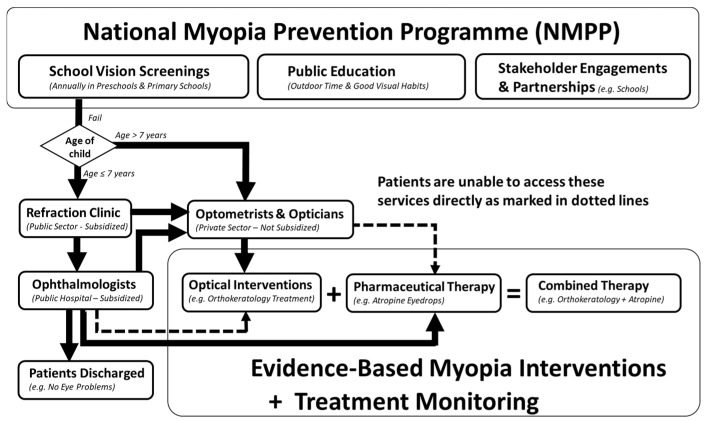
Schematic representation of the National Myopia Prevention Programme (NMPP), referral pathways of the school vision screening programme, and the current role of ophthalmologists, optometrists, and opticians in managing children with evidence-based myopia interventions.

Singapore is one of the few countries worldwide that has implemented government-instituted policies on children myopia prevention. Recognising the disease burden of myopia since 2001, the National Myopia Prevention Programme (NMPP) comprises school vision screenings and public education campaigns that promote outdoor activities and good eyecare habits to prevent myopia or delay its onset (Figure 1) [4].

School vision screening serves as the first line of defence to combat myopia. While passing the vision screening can sometimes give parents a false sense of assurance, it helps to identify school children with myopia and other vision problems if they have not already attended an eye examination. Children with early-onset myopia are more likely to develop high myopia [11], so it is important for them to be diagnosed early and monitored carefully. Poor vision can also affect the child’s visual development (e.g., amblyopia) [12,13,14,15,16], hinder learning [17,18,19], and negatively impact mental health and quality-of-life [18].

Public health campaign have been targeting families and children to spend 8 to 15 h outdoor time each week to prevent myopia [11], because outdoor environments helps to release retinal dopamine, a neurotransmitter which inhibits myopic eye growth [20,21], whereas indoor environments tend to deprive high spatial frequencies resulting in myopic eye growth [22]. While outdoor activities are generally cost-effective and have led to improved outcomes evidenced by the reduced prevalence of myopia amongst primary school children [23], the NMPP has minimal impact amongst secondary school children [24]. Recent research suggests that outdoor activities have less effect on older children, especially when they are already myopic [25], and Singaporean teenagers are already spending an average of 3.2 h each day on outdoor activities [26]. Hence, it is crucial for all children to be able to access evidence-based myopia interventions rather than relying entirely on outdoor activities.

With the two decades of the NMPP, there is currently a pressing need for policymakers to review and update its current policies in order to serve the needs of future generations of Singaporeans. Firstly, a large volume of myopia research has emerged worldwide in the past decade demonstrating promising outcomes from evidence-based myopia interventions. Secondly, outdoor activities alone may not be sufficient to address the rapid myopia progression in children who are already myopic [25]. Thirdly, there is a need to consider the rising healthcare costs of myopia and its burden on the country’s healthcare system [10]. Fourthly, it is important to plan for future healthcare manpower needs in ophthalmology and optometry and to explore various models of care in order to cope with the rising workload from the disease burden [27,28]. Thus, it is critical for Singapore to ramp up its efforts in controlling the progression of myopia.

### 1.2. Evidence-Based Myopia Interventions

There has been an emergence of many evidence-based myopia interventions in the past two decades. These can be broadly classified as: (1) optical interventions, (2) pharmaceutical therapies, and (3) combined therapies [29].

Optical interventions are the fundamental treatment of myopia, as they provide clear vision and are able to control the progression of myopia. This comprises orthokeratology treatment and specific spectacle and/or contact lens designs and technologies for myopia control (e.g., spectacle lenses with highly aspherical lenslets or HALs). While the costs are high, these interventions have been found to be cost-effective in controlling the progression of myopia in children [30]. In contrast, single-vision lenses are able to provide clear vision but do not have any effect in controlling myopia progression in children [31].

Pharmaceutical therapies comprise low-dose atropine eyedrops ranging from 0.01 to 0.05% to as high as 1% dosage in some situations. While 0.05% is regarded the optimal [32,33] and cost-effective dosage [30], local public hospitals tend to initiate treatments with 0.01% [34] and vary the dosage and/or frequency of administration according to patient responses, side effects, and/or ages. Approximately 3–7% of children on low-dose atropine may develop allergic conjunctivitis, and 30–40% may need photochromic spectacles due to light sensitivity and/or glare [35]. However, progressive addition lenses are seldom required as only a dosage above 0.02% is expected to induce symptoms and/or clinical signs of insufficient accommodation and/or excessive pupillary dilation [36].

Combined therapies, such as the combination of 0.01% atropine and orthokeratology treatments, are widely regarded most effective due to their “synergistic” or “additive” effects [29,37,38,39]. It is also possible to combine atropine with myopia control spectacle lenses or contact lenses [40]. Another advantage is the opportunity to use 0.01% dosage, which averts the use of a higher dosage and its side effects. In addition, there is increasing research supporting behavioural approaches (e.g., environmental modifications), repeated low-level red-light therapy [41], and the clinical management of binocular and accommodative functions (e.g., vision therapy) [42,43].

### 1.3. The Current Situation in Singapore

Given the emergence of evidence-based interventions in the past two decades, there is now compelling reasons to review the current public health policies and its associated inequities in relation to myopia control in children. Research suggests that the prevalence of high myopia can be reduced by nearly 90% if the myopia progression rate is halved [2]. Quoting the Singapore Health Minister, Mr Ong Ye Kung, who spoke in parliament on 11 May 2021, *“By reducing myopia progression rate to 50%, through the combined use of pharmacological and optical therapies, the prevalence of high myopia could potentially be reduced further by up to 90%”*. Thus, it is of public interest to explore how the current healthcare system can be reviewed to encourage the uptake of these evidence-based myopia interventions and to prepare for the future needs in managing the disease burden of myopia.

Based on practitioner surveys in Asia, combined therapy consisting of orthokeratology treatment and atropine only contributes 5.3% of the current treatments [44]. If only single therapies are considered, uptake remains poor as each of the individual approaches only account for 14.6% (atropine) and 8.7% (orthokeratology), respectively [44]. Other single therapy options with myopia control spectacles and other types of contact lenses are 16.8% and 5.7%, respectively. Uptake of combined therapy is poor due to treatment costs (33.2%), availability (24.1%) [44], and structural barriers such as regulatory restrictions, time constraint, and inconvenience. While single therapies can be offered to patients, the main approach by the majority of practitioners still involves single-vision spectacle lenses (32.2%) and other types of spectacle lenses or contact lenses (9.7% and 7%, respectively), which are unlikely to control myopia progression [44].

**Figure 2:**
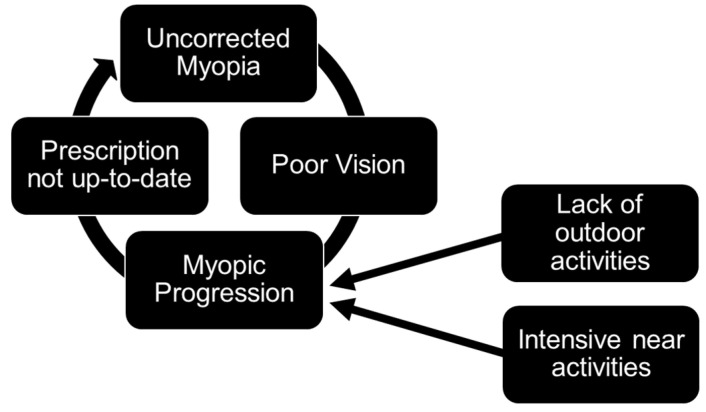
A vicious circle of myopia progression due to under- or un-corrected myopia, which may disproportionately affect low-income families and widen health inequities.

In Singapore, optometrists are licenced, trained, and skilled in providing primary eyecare and evidence-based myopia interventions, including the prescribing of all kinds of optical inventions and the early detection of eye diseases. This is crucial to patient care because those who are not appropriately managed with optical interventions are at a greater risk of developing high myopia. As supported by two systematic reviews, the current clinical recommendation advocates the full correction of myopia, whereas under-correction causes faster myopia progression [45,46]. If myopia is left undetected or if myopia remains under- or un-corrected (e.g., spectacle prescriptions are not up-to-date or if children do not wear the spectacles regularly), vision will not only be blurred, but it can also accelerate axial elongation in rapid myopia progression [45,46]. This can negate the benefits of both pharmaceutical therapies and outdoor activities since the loss of high spatial frequencies is similar to the retinal mechanisms of form-deprivation myopia, which disrupts retinal stop-signalling pathways for axial elongation [22]. In the long term, this can potentially result in a vicious circle of deteriorating vision that deepens health inequalities (Figure 2). Therefore, it is important for spectacles and/or contact lenses to be prescribed or dispensed appropriately [43] and for children to be monitored by optometrists in the community [11].

### 1.4. Barriers Affecting the Uptake of Evidence-Based Myopia Interventions in Singapore

This present policy review has identified six key barriers:

Firstly, current policies lack the provision for optometrists to prescribe medications and/or to use topical diagnostic drugs for cycloplegic refraction, pupil dilation, and local anaesthesia for checking intraocular pressure and/or fitting contact lenses. Thus, there is limited scope of practice for them as a primary eyecare provider [47], and this limits their ability to render pre-myopia risk assessments for children since diagnostic drugs are needed to properly evaluate the “hyperopic reserve” in relation to the child’s age [48,49]. For this reason, optometrists in Singapore are currently unable to offer the full range of evidence-based myopia interventions and services to their patients.

Secondly, combined therapy can be inconvenient and time-consuming due to multiple visits to optometrists and ophthalmologists. Although regulatory requirements are intended to safeguard public safety, low-dose atropine eyedrops rarely have significant side effects but can only be supplied through “in-clinic dispensing” by ophthalmologists [50]. Hence, multiple ophthalmological visits are required in addition to existing visits to the optometrist to monitor treatment effects, eye health, and myopia progression.

Thirdly, consumer decision making tends to be guided by personal beliefs, preferences, attitudes, and practical concerns by practitioners [44,51], such as inconvenience, regulation, and cost, rather than evidence-based approaches. It is also conceivable that myopia control spectacle lenses are influenced by commercial determinants of health [52], driven by consumer advertising and retail sales practises instead of evidence-based clinical judgements and/or indications following a comprehensive eye examination. This is due to the country’s widespread availability of “refraction-only” eye examinations to children above the age of 7 years [53], which is often conducted as part of the retail sale of spectacles. The term “refraction-only” refers to the testing of refractive error as a “standalone service” without a complete assessment of the eye health and/or other visual functions according to the World Council of Optometry (WCO) [54]. This falls under Level 1 of the 2022 WCO Competency Framework due to the omission of optometric assessments that are expected from Level 2 [55]. In contrast, Levels 3 and 4 entail the investigation of eye conditions and pharmaceutical prescribing, respectively [55]. This framework is recently updated to align to the WHO Global Competency Framework to guide the standards for education and practice for health workers in primary care [56].

Fourthly, treatment costs are a major concern in Singapore and in Asia (33.2%) [44], due to the significant out-of-pocket expense. While universal healthcare coverage (UHC) for Singaporeans covers up to 80% of healthcare costs, these subsidies do not cover optical interventions as the services are mostly rendered by optometrists in private practice or optical shops, and only a small proportion of patients are able to claim these expenses from employer health benefits and/or private insurance. Comparatively, Australasia (12.9%) and North America (16.6%) are less concerned with treatment costs because UHC and insurance cover optometry visits [44].

Fifthly, there is a lack of advertising regulation to safeguard the consumers from misinformation about myopia control, because there are many unproven commercial approaches, such as pinhole glasses, devices, alternative remedies, and eyesight improvement workshops. Some parents may hold certain beliefs, feelings, or attitudes towards evidence-based approaches. For example, parental disapproval with spectacles as nearly half of those surveyed felt that “glasses make their vision worse” or “perfect vision is unnecessary”. In addition, nearly half of the free spectacle vouchers to children from low-income families are unutilised each year due to some of these reasons [57], and some of them may prioritise outdoor activities rather than wearing spectacles.

Finally, there is a general public perception that evidence-based myopia interventions can only be accessed from public hospitals due to the high public trust of the healthcare system and it is government-subsidised. Even though the optical industry is regulated, there is still a lack of mandate to institutionalise optometrists as the primary eyecare provider. As optometrists have limited scope of practice, some patients may delay optical interventions while waitlisted at public hospitals, and some may be lost to follow-up, making it difficult to keep track of non-compliance.

### 1.5. Rationale of Pharmaceutical Prescribing Privileges for Optometrists

Childhood myopia is typically managed by optometrists in the community amongst many other eye health and vision conditions. The proposal of pharmaceutical prescribing privileges for optometrists is primarily motivated by the premise of improving access to services and reducing the direct and indirect costs of combined therapy. Although myopia progression is a challenge mainly in the paediatric population, there are concerns of elevated risks for myopia-related eye disease that would increase the disease burden in the future. Children from low-income families are most vulnerable [19], and they are most at risk for these issues to become entrenched into adulthood, impacting work productivity and future career prospect [58].

With the ageing population and manpower constrains within the public healthcare system, there has been suggestions for task-shifting from ophthalmologists to optometrists to handle the stable eye conditions and issues that do not require urgent referrals [47]. Nearly a quarter of optometrists in Singapore have attained additional qualifications beyond the basic requirement for licensure, but they still do not have pharmaceutical prescribing privileges. For this reason, a proportion of patients still have to be referred to the ophthalmologists for dilated eye examination (mydriasis), cycloplegic refraction, and atropine eyedrops, even though these services can be safely and proficiently conducted by optometrists as evidenced by the experience from other countries.

In relation to public health, optometrists have helped to triage urgent cases in pilot screening programmes in collaboration with hospitals and specialist centres within the public health system, which comprise three integrated clusters (i.e., National Healthcare Group (NHG), Singapore Health Services (SingHealth), and National University Health System (NUHS)). However, the lack of pharmaceutical prescribing privileges for optometrists meant that non-urgent cases may still be referred subsequently. In other healthcare professions, Singapore’s Ministry of Health (MOH) has introduced advanced practice roles for pharmacists and nurses, which allowed them to legally prescribe medications under Collaborative Prescribing agreements [59]. Legislated by the Healthcare Services (Collaborative Prescribing Service) Regulations 2023, these advanced roles have improved patient care, perceived patient outcomes, patient access, and productivity, have reduced workloads, and have led to greater job satisfaction amongst those professions [59].

In 2017, MOH launched the “Beyond Healthcare 2020” strategy, which was intended to “bring healthcare closer to home”, promote healthy living, and ensure value. In line with this strategy, it is beneficial for primary care and chronic conditions to decentralise from the public healthcare system into community settings (“beyond hospital to community” and “beyond quality to value”). Thus, an extension of pharmaceutical prescribing privileges to optometrists can make primary eyecare more accessible, and there is greater opportunity for optometrists in the community to cooperate and co-manage patients. In the context of childhood myopia, this strategy is also likely to improve access, lower the cost of evidence-based myopia interventions, and provide greater value to the patient.

### 1.6. Aims and Objectives

In view of the unique healthcare landscape and growing prevalence of myopia in Singapore, this policy review investigates the policy-level challenges in community-level interventions of childhood myopia. While optometrist prescribing privileges are proposed to reduce the barriers to evidence-based myopia interventions and enhance patient co-management between optometrists and ophthalmologists, its potential benefits, drawbacks, and related policies have not been analysed yet. The aim is to conduct a policy review and analysis to determine the most viable public policy option to reducing the barriers in accessing evidence-based myopia interventions.

## 2. Methods

A systematic search of the literature was conducted on the PubMed database in August 2024 to gather relevant evidence from peer-reviewed articles of optometrist prescribing privileges and the various models of co-management between optometrists and ophthalmologists from other countries. Search terms were combined with Boolean operators (“AND” and “OR”) to find relevant matches from the titles, abstracts, and keywords, if available. As optometrists fall under the category of “non-medical prescribers” in many countries, the search terms are entered as follow: (“non-medical prescrib*” OR “nonmedical prescribe*”) AND (“optometrist” OR “optom*”)>, and <(“independent prescrib*” OR “supplementary prescrib*”) AND (“optometrist” OR “optom*”)>, where the asterisk in “prescrib*” covers the terms, such as “prescribing” and “prescriber”. Relevant articles with accessible full texts in the English language are included, whereas conference abstracts, dissertations, and book chapters are excluded.

Of the evidence gathered, two policy options are identified in this policy review as they have been found to work in other countries. The current policy and the two policy options were analysed using the Centers for Disease Control and Prevention (CDC) Policy Analysis Framework to explore their potential benefits and drawbacks in terms of its public health impact, feasibility, and economic and budgetary impacts, and evidence-based policy solutions and gaps in the evidence base are identified. The scope of the analysis is on pharmaceutical prescribing privileges in relation to children myopia management only, so the treatments of other myopia-related disease complications are not included. In addition, this policy review and analysis does not cover diagnostic pharmaceutical agents as there is already existing evidence supporting its use by optometrists.

The public health impacts of each of the policies were analysed in the domains of operational efficiency, treatment costs, and reducing the barriers to treatments, and their feasibility were analysed in the context of the existing regulations, guidelines, and infrastructure, addressing stakeholder concerns, its implementational challenges, timeline, and costs. Local data are used to support the policy analyses where available, and reference data from other countries and/or other diseases are also used. The economic and budgetary impact of the policy options were analysed based on the annual treatment costs to each individual patient because these costs are largely out-of-pocket with the exception of spectacles, which may be defrayed using vouchers eligible to low-income families [57].

The estimated costs of treatments and medications are based on market research under the following categories: (1) optical shop, (2) optometry clinic, (3) private ophthalmology clinic, and (4) public hospital. Due to the mixed healthcare market, there is a wide variety of choices and options for the patients, so variations in treatment costs tend to depend on specialised offerings, cost structures, profit margins, and operational costs of the individual practices. For clarity, it should be noted that the term “public hospital” is used throughout this policy review because the three integrated clusters have been restructured to operate these hospitals as private companies but are wholly owned by the Singapore government. A distinction is made between optical shops (i.e., retail-based) and optometry clinics (i.e., healthcare-based) because they differ in terms of breadth and depth of service offerings and fee structure. For example, “refraction-only” eye examinations are often rendered by either refracting opticians (children above the age of 7 years) or optometrists (children of any age) as part of the retail of spectacles, whereas comprehensive eye examinations tend to be provided only in clinical settings or in optometry clinics [53].

Computation of the annual costs of consultations are based on four quarterly visits, as this would be the same frequency as orthokeratology-related follow-up visits and also the same frequency where the prescriptions of atropine are filled. Quarterly visits are reasonable estimates considering that the minimum treatment monitoring intervals is six months according to the International Myopia Institute (IMI) Clinical Management Guidelines [11], but typically more frequent in the initial stages of treatment or if treatment responses are poor (<1 to 3 months) and may be less frequent if the ophthalmologist refills the prescription over-the-counter. Reflecting real-life situations, costs of treatments and consultation fees are itemised for public hospitals, private ophthalmology clinics, and optometry clinics, but these fees are not separable for optical shops as the cost of consultation and services tend to be factored into the retail purchase.

In the policy analysis, it is assumed that optometrists in the community do not increase the charges during independent prescribing and that the ophthalmologist at public hospitals and private clinics would levy S$100 and S$150 per visit, respectively (in Singapore dollars, S$) due to fee-sharing under the supplementary prescribing arrangement. This levy assumes a reasonable and fair negotiation between two parties, which can vary or be renegotiated according to open market forces, but it cannot be presumed to be in non-monetary terms, such as increased referrals or gift gratification, due to its unaccountability and ethical concerns.

## 3. Results

This policy review examined the current public health policies and its associated inequities in relation to myopia control in children. Of the 25 papers found from the literature search, 12 studies satisfied the inclusion criteria and were included (Appendix A). Eight studies were excluded due to its irrelevance to the topic, four duplicates were removed, and one is an erratum of a study that has already been included.

### 3.1. Public Health Impact of Pharmaceutical Prescribing Privileges for Optometrists

As identified from the literature search, two policy options have been found to be feasible in other countries. These policy options includes “independent prescribing” and “supplementary prescribing” [60,61], where the former entails autonomous clinical decision making for optometrists in the community to write medical prescriptions that can be filled at pharmacies, and the latter entails a co-management framework under the supervision of ophthalmologists to supply medications. The latter would require co-management contracts to be established with ophthalmologists, either within public hospitals or private ophthalmology clinics. These policies are compared with the status quo, as summarised in Table 1.

According to reference data from the UK, optometrist prescribing privileges can increase patient access by 20–50% [62], since a large number of optometrists are readily available in the community. Both policy options are expected to improve access to the full range of evidence-based myopia interventions by: (1) streamlining treatment processes so that all tests can be completed within one visit, (2) reducing treatment costs, as optometry visits tend to cost less than ophthalmology appointments as well as eliminating duplicated testing, and (3) reducing the barriers concerning wait times and inconvenience.

While supplementary prescribing leverages on the expertise of ophthalmologists in rendering comprehensive care, independent prescribing is more favourable because (1) it is more cost efficient, (2) it substantially reduces healthcare costs, (3) it is more likely to reduce health inequity, and (4) it is more likely to reduce the workload of ophthalmologists [27,62]. If the situation remains the status quo, the public health impact of improving access to the full range of evidence-based myopia interventions will not be realised because treatment availability is a major concern in Asia (24.1%) [44]. Comparatively, Australasia (7.9%), Europe (10.5%), and North America (11.0%) have less concerns about treatment availability due to the existing pharmaceutical prescribing privileges of optometrists [44].

Many of the current challenges of myopia prevention and management are in the level of the community, which tend to involve optometrists. Since optical interventions should precede pharmaceutical therapies, it is not tenable for public hospitals to address the public health challenges with atropine alone without co-managing the patients carefully with optometrists in the community. As there are no existing frameworks in Singapore for the co-management of childhood myopia, it is possible that parents are offered conflicting advice or misinformation. If the situation remains the status quo, the concerns are the following: (1) delayed interventions, (2) lengthy and time-consuming treatment processes, (3) high treatment costs and overall healthcare costs, (4) poor uptake due to wait times and inconvenience, (5) poor treatment compliance if patients are unable to follow-through the appointments, [63] and (6) health inequities if treatments remain unaffordable [19].

### 3.2. Feasibility of Pharmaceutical Prescribing Privileges for Optometrists

Both independent and supplementary prescribing can help to improve public access to the full range of evidence-based myopia interventions as optometrists are already trained to diagnose and manage myopia and have existing equipment [47,62]. However, the feasibility of each of these policy options requires stakeholder input, including ophthalmologists, consumer organisations, and policymakers, to address their concerns carefully. It also entails legislative changes, regulation changes, and government fundings in order to implement these policies.

To address the public concerns of independent prescribing, it may be necessary to implement additional training, certification, and prescribing guidelines to ensure that the optometrists are competent in their new scope of practice. While optometrists have demonstrated various advanced competencies [60,64,65,66,67], these additional training, certification, and prescribing guidelines would help to allay the following concerns: (1) compromised treatment outcomes due to less extensive medical training [47], (2) patients may be at risk of potential misdiagnosis, or misuse of the medication [68], (3) safety issues if the optometrist is poorly trained, incompetent, or unethical [68], and (4) public perception that they are receiving lower quality-of-care [69]. While these concerns may be valid, independent prescribing is already an existing scope of practice of optometrists in many countries as they manage eye diseases and refer to the ophthalmologist only when needed. This approach has proven to be successful in the US, UK, Canada, New Zealand and Australia without compromising outcomes [64,70,71]. The additional training can also help in upskilling and improving their competencies [65,72], as it is possible to achieve good clinical concordance between optometrists and ophthalmologists [73].

In contrast, the concept of supplementary prescribing is similar to Collaborative Prescribing that is already in place for advanced practising nurses and pharmacists in Singapore [59], although this may entail additional supervisory co-management arrangements and administrative work. Unlike Collaborative Prescribing where the co-management arrangements are typically within the same organisation, there will be a need to establish commercial contractual agreements under supplementary prescribing because optometrists and ophthalmologists do not usually practice within the same organisation. Hence, there may be potential concerns such as: (1) financial and ethical considerations, (2) possible conflict of interests, as commercial contractual agreements may lead to over-referrals [74], (3) reduced efficiency of the treatment process, and (4) increased treatment costs due to the extra oversight by ophthalmologists. These drawbacks may explain why supplementary prescribing is unpopular among non-medical prescribers in the UK [61].

In terms of implementation, optometrists are likely to find independent prescribing more favourable [61], and would proactively complete the training and certification due to quest of knowledge and confidence [72], and their vested interests to increase their scope of practice. A recent study showed that UK hospital-based optometrists gained advanced competencies across eight domains of clinical practice when they have independent prescribing privileges [64], and they are able to offer a variety of clinical procedures and/or interventions independently [65].

Within community settings, private practising optometrists from these aforementioned countries are already treating eye conditions independently with successful working relationships with ophthalmologists, and effective referral systems [70,71]. Such collaborations also exist currently in Singapore, where public hospitals outsource initial evaluations, stable eye conditions and post-surgical refraction to optometrists in the community [75]. During the COVID-pandemic in 2020–2021, these arrangements helped to eliminate unnecessary visits to public hospitals and minimised the spread of coronavirus. It also substantially reduced the time taken for initial evaluation by 86%, and a 67% reduction in complaints regarding the long waiting times [75,76].

Although pharmaceutical prescribing privileges for optometrists has advantages, pushbacks are anticipated from ophthalmologists with traditional mindsets, opposing views, and vested interests [77]. Resistance to changes can also be due to the fear of uncertainty, loss of control and anxiety. Other stakeholders may perceive the quality-of-care to be poorer even though treatment outcomes are comparable with ophthalmologists [70,71]. This is not surprising due to the lack of public awareness on the specific roles of each profession, and this may be incorrectly perceived as a hierarchy [78]. While negative attitudes and hierarchical views can affect the uptake of optometry services, 82% of patients surveyed from a public hospital indicated willingness to use the outsourced optometry services on their next visit [75,76]. Furthermore, patient satisfaction achieved averagely 4/5 on the Likert scale in a Collaborative Prescribing model by nurses [79].

Since independent prescribing is a new concept in Singapore and the general public is still unfamiliar, it is essential for optometrists to communicate facts properly to allay the misconceptions amongst stakeholders. If independent prescribing is implemented, this may entail an estimated timeline consisting of the following: (1) 6 months for pilot study, (2) 6 months for legislative and regulatory amendments, (3) 6 months for additional training and certification, and (4) 6 months for developing guidelines. In contrast, the implementation of supplementary prescribing is subject to contractual agreements through commercial decisions. To align with patient’s interests, a framework can help to guide the co-management arrangement, including how the care and fees should be shared between the parties involved. In the UK and Australia, this has worked well from reimbursements by the National Health Service and Medicare, but this current policy option involves payments out-of-pocket, so it is possible for treatment costs to spiral under commercial influence if a proper framework is not in place.

Past experiences in the UK have shown supplementary prescribing to be less favourable among non-medical prescribers due to the increased requirement to co-manage the patients with medical doctors [61]. Sharing of medical records can be problematic due to the possibility of incompatible electronic databases, and additional administrative duties can reduce efficiency and delay treatments. If ophthalmologists need to intervene, patients may be confused, and tests may be duplicated. However, innovative approaches may help to mitigate these operational challenges (e.g., telemedicine). Furthermore, this policy option is vulnerable to stall or terminated if there are (1) contractual disagreements [80] due to lack of consensus [81] or vested interests [77], (2) negative feelings due to the hierarchical system [82], (3) strained relations in situations of unfair advantage, and (4) if it is inconsequential for ophthalmologists to support optometrists’ agenda.

While a two-year timeline is estimated for its implementation and policy enactment, a detailed roadmap should take into account early consultations and dialogues with the respective stakeholders who may be directly and indirectly impacted by the proposed changes. The implementation of independent prescribing for optometrists would be similar to Collaborative Prescribing for advanced practising nurses and pharmacists, and the “Guidelines for The Implementation of Collaborative Prescribing Services” published by the MOH in 2018 are already in place. However, these policies and guidelines must be expanded to include optometrists in the community because the current arrangements are still confined within hospitals, polyclinics, and nursing homes. To achieve this, an advisory committee can be formed to develop strategies and apply the lessons learnt from the successful case studies of current Collaborative Prescribing models as well as by gathering feedback from stakeholders to identify issues and challenges. For example, safety concerns can be addressed with training and certification, and a restricted formulary can also be implemented such that only medications relevant to optometry practice (e.g., low-dose atropine eyedrops, cycloplegics, mydriatics, and local anaesthesia) are included. Efforts should also be focused on communication, building trust, and fostering positive stakeholder relationships.

### 3.3. Economic Impact of Pharmaceutical Prescribing Privileges for Optometrists

Myopia puts substantial financial burden on patients and their families in Singapore due to the out-of-pocket expenditure on optical interventions and optometry services [83]. While considering evidence-based myopia interventions, combined therapies are particularly expensive due to the costs of optical interventions in combination with pharmaceutical therapies and the multiple separate visits with optometrists and ophthalmologists. In this present analysis, the annual treatment costs of both policy options are compared with the status quo (Table 2), and each of the estimated costs are itemised for each patient accordingly (Table 3).

**Figure 3:**
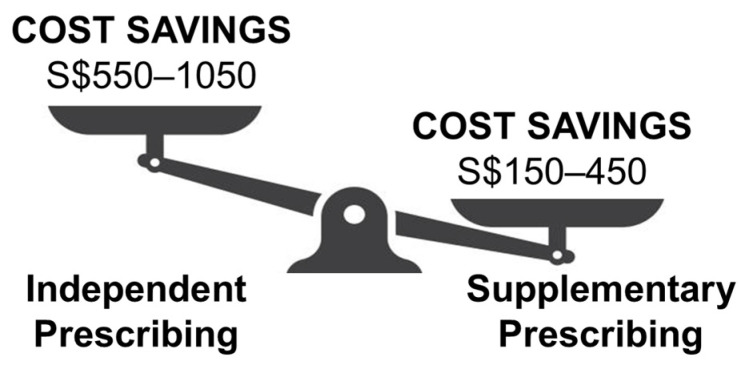
Annual treatment cost savings for each patient in Singapore dollars (S$) when comparing between independent and supplementary prescribing by optometrists.

Direct savings are found to be 16–25% for independent prescribing and 5–10% for supplementary prescribing, which vary according to the provider category, and there are also indirect savings from fewer travels and time taken from work to attend these appointments [83]. Estimated potential cost savings from independent prescribing are more substantial than supplementary prescribing (Figure 3), because fee-sharing is not involved, although there may be variations depending on its uptake and system level changes. The estimated cost of each optometry consultation from this analysis is comparable to previous reports of S$140 per visit [84], which is substantially more cost-effective than frequent visits to the ophthalmologists. In addition, patient co-management arrangements and regulatory compliance for supplementary prescribing are expected to increase system level costs, which may eventually be passed down to the patient’s expense if such costs are not subsidised by the government.

### 3.4. Budgetary Impact, Disease Burden, and Health Inequality

Given that medication dispensing is not involved, there are no system level costs in independent prescribing, but government fundings may be needed for implementing and evaluating the pilot programme, and for setting-up the training course. In terms of ease of implementation, these certification and training courses can be added to existing courses for optometrists funded by government grants, such as Graduate Certificate programme from the National University of Singapore. Fees for independent prescribing certification can be at the optometrist’s expense, since the expansion of the scope of practice may increase their income. Myopia deserves urgent attention as the country’s healthcare costs are substantially higher (nearly one billion Singapore dollars) compared to other health conditions, such as Parkinson’s Disease (S$32–57M), Chronic Obstructive Pulmonary Disease (S$12M), and Acute Angle Closure Glaucoma (S$0.3–0.6M) [84]. Substantial benefits to public health are anticipated with evidence-based myopia interventions, because it can potentially half the rate of myopia progression and reduce the prevalence of high myopia by 90% [2].

The disease burden of myopia is expected to increase with the country’s ageing population and increasing risk of myopia-related complications. While disease burden can be mitigated with independent prescribing by optometrists [85], referral of these eye diseases can still continue to soar due to the rapidly ageing population [86]. Early treatment will be more cost-effective [87], as shown by the incremental cost-effectiveness ratio (ICER) of S$1400 per quality-adjusted life years (QALY) for 0.01% atropine versus S$18,000–345,000/QALY for treating pathologic myopia [87,88,89]. However, the current approach in myopia control is still insufficient because 20–30% of atropine users need a higher dosage, causing side effects such as accommodative dysfunction and photosensitivity [29]. Treatment of these side effects indirectly escalate healthcare costs [90], but these costs can be averted if a higher dosage can be avoided with combined therapy. This is because low-dose atropine (0.01%) has minimal or no side effects, as only 30–40% of them are likely to need photochromic spectacles [35], and the optometrists are in the best position to manage these minor visual symptoms if they arise during follow-up visits.

Myopia, if left uncorrected, can result in a loss of productivity by 3.1–18.7% as a proportion of the gross domestic product (GDP) [58,91]. There are high risks for developing eye complications, such as macular degeneration [6], retinal detachment [7], glaucoma [8], and blindness [9], which carries significant lifetime costs. With the country’s ageing population [92], and the current shortage of ophthalmologists [27], treatments of these complications will become a huge burden on the Singapore healthcare system. By granting independent prescribing privileges to optometrists, it will alleviate the workload of ophthalmologists, so that they can focus on more complex conditions [93,94]. For example, optometrists in the community have helped to reduce the burden on public hospitals as demonstrated during the 10-week COVID lockdown in England [66] and Wales [93], and as much as 66% of patients from England’s public hospitals can be managed by optometrists in the community [94]. In addition, the cost savings on services rendered by optometrists can help to alleviate the patients’ financial burdens, which tends to disproportionately affect low-income families, widen health inequities, and limit access to treatments.

With the careful consideration of the public health impact, feasibility, and economic and budgetary impacts of these two policy options, this present policy review and analysis favours independent prescribing for optometrists because its substantial benefits outweigh the drawbacks in implementing this policy in Singapore.

## 4. Discussion

This policy review investigated the policy-level challenges in community-level interventions of childhood myopia in Singapore. The policy analysis has carefully examined the potential benefits and drawbacks of optometrist prescribing privileges and related policies to reduce the barriers to combat childhood myopia and explored the enhancement of patient co-management between optometrists and ophthalmologists to reduce the barriers to evidence-based myopia interventions.

### 4.1. Key Policy Solutions and Recommendations

This policy review supports independent prescribing for optometrists because childhood myopia can be managed successfully at the community level. Optometrists play a pivotal role in prescribing the appropriate spectacles and/or contact lenses, which are frequently necessary before exploring other evidence-based myopia interventions. An increased scope of practice is likely to benefit public interests substantially, and they are suitable for independent prescribing given their knowledge, expertise, and training in utilising specialist diagnostic instruments. If atropine eyedrops are prescribed, optometrists are also in the best position to manage side effects related to visual symptoms instead of visiting the hospital as further training can allow optometrists to manage patients safely in the community.

Regardless of independent prescribing or supplementary prescribing, treatment processes can be substantially streamlined, such that patients can receive diagnoses, treatment plans, and medications within one visit. This not only offers convenience to patients, but also improves access and reduces treatment costs, which helps to encourage early interventions, improves treatment compliance, reduces loss to follow up, and reduces wait times in public hospitals. Collectively, this is expected to reduce the risks of the population developing high myopia, disease complications, and/or blindness, so as to mitigate the disease burden and future healthcare costs. Thus, pharmaceutical prescribing privileges for optometrists can substantially improve public health outcomes.

Independent prescribing is the preferred policy option because optometrists are already licenced and government regulated and can significantly reduce out-of-pocket expenditure. This can be easily implemented with additional training, certifications, regulations, and by prescribing guidelines for optometrists as well as legislative and regulatory changes. In contrast, supplementary prescribing lacks predictability and stability to support its long-term viability and continuity, and the fee-sharing structure and commercial interests have controversial ethical concerns that can result in over-referrals. Despite these shortcomings, ophthalmologists and optometrists should still collaborate under an independent prescribing framework to provide the best possible care to patients through professional referrals that are unimpeded by commercial interests.

### 4.2. Additional Recommendations and Considerations

While this policy review focused primarily on prescribing privileges for optometrists, there are four additional considerations that are important to the nation’s efforts in combating myopia in children:

Firstly, collaborations between optometrists in the community and public hospitals is currently lacking, so various models of care should be explored to facilitate partnerships without compromising the quality of care. Pharmaceutical prescribing privileges for optometrists can help to foster partnerships with public hospitals as the additional training, certification, and accreditation will improve patient care. Although some positive developments are observed in pilot projects involving optometrist-led co-management of stable eye conditions, primary and community care is generally less developed compared to acute and secondary care sectors due to the thin operational budgets and margins within the public hospital or integrated cluster [95]. Given the insufficient capacity for public hospitals to collaborate with the private sector [95], it is likely that such collaborations will perform better through a national mandate by the Ministry of Health that institutionalises optometrists as the primary eyecare provider rather than collaborating under the auspice of public hospitals.

Secondly, there is a necessity to encourage evidence-based myopia management and to regulate consumer advertising of commercial products aimed at children myopia management. While pharmaceutical prescribing privileges for optometrists can widen their scope of practice and manage myopia more comprehensively, there is an abundance of product choices driven by consumer advertising and retail sales practises that may confuse the general public. There is widespread availability of “refraction-only” eye examinations, which is insufficient to guide evidence-based clinical decision making. Thus, this policy review recommends regulatory changes to ensure that comprehensive eye examinations are conducted prior to the initiation of any myopia intervention. Due to the variability of optometry qualifications, national accreditation and specialisation is recommended, such that pharmaceutical prescribing privileges are accorded only to those who demonstrate competency. Professional trainings on myopia control and national accreditations should also be free from commercial influence. Furthermore, advertising regulation on commercial products would safeguard consumers from misinformation about myopia control.

Thirdly, there is urgent necessity to enhance public education and to curb misinformation. Patients may not heed professional advice due to personal beliefs, feelings, or attitudes towards spectacles [96], contact lenses [97], and atropine eyedrops [98]. Given the evidence that nearly half of the children who needed spectacles are not actually wearing them [57], it may be necessary to emphasise on the importance of spectacle compliance due to their risks of myopia progression [45,46]. Similarly, myopic children who wear contact lenses and those who use atropine eyedrops often need to follow specific instructions to optimise their treatments, and failure to do so, particularly with atropine, can lead to no effect or rebound effect when patients discontinue its usage without careful monitoring [97,98]. Thus, it is particularly advantageous for optometrists in the community to render the necessary care since they are easily accessible to the public compared to hospitals.

Fourthly, it is important establish a formulary for optometrists that takes into account of their full scope of practice, because they need access to a range of appropriate pharmaceutical agents in order to render their services effectively. To combat myopia, they should be able to: (1) assess the risks of myopia (or pre-myopia), (2) provide evidence-based myopia interventions, (3) monitor the interventions, (4) detect myopia-related diseases, and (5) assist in collecting epidemiological data to strengthen myopia surveillance and research. For example, they are presently unable accurately assess risks in pre-myopia because they do not have access to cycloplegic eyedrops to accurately evaluate the “hyperopic reserve” [48,49]. This may affect the monitoring of the interventions in some situations, and it may be challenging to detect myopia-related diseases without access to mydriatic eyedrops. Furthermore, they may not be able to fully contribute to myopia surveillance because non-cycloplegic refraction tend to result in an overestimation [99,100,101], and misclassification of the epidemiological data [102].

### 4.3. Strengths, Limitations, and Gaps in the Evidence

This policy review is useful to policymakers in decision making and to allocate resources strategically. The strength of this paper is the use of a broad range of local data to support the cost-savings from independent prescribing, despite having uncertainty about the specific expenditure commitment of its implementation. While the feasibility of both policy options requires stakeholder input, including ophthalmologists, consumer organisations, and policymakers, the policy analysis favours independent prescribing for optometrists because many of the anticipated pushbacks and concerns can be addressed.

The method of appraisal allows policy options to be systematically analysed based on the relevant factors and evidence, and an appraisal of the quality of evidences and data from this policy analysis is summarised in Appendix B. Due to the focus on policy options on the pharmaceutical prescribing privileges for optometrists, this policy review may not fully address the societal or ethical considerations of patient co-management. Representative data from other countries are used to justify independent prescribing based on specific eye conditions, which may not be generalizable to support the prescribing of other medications.

Primarily, the representative data from this present policy analysis are derived from the UK, because the National Health System demands high standards in public health reporting from peer-reviewed publications as they tend to take a cautious approach while introducing pharmaceutical prescribing privileges amongst non-medical professionals. The adoption of optometrists prescribing privileges in the UK are also more representative of the situation in Singapore as Collaborative Prescribing agreements in Singapore have only recently been introduced to pharmacists and nurses with advanced practice roles. In contrast, optometrists in Australia and New Zealand have had pharmaceutical prescribing privileges for 15 years and are able to prescribe atropine eyedrops to control the progression of myopia in children [103], and optometrists in USA have a long history of these privileges, which not only covers topical eyedrops but also oral medications in some states [104]. In these countries, the healthcare financing systems differ from Singapore, so these models may not be directly transferrable to Singapore’s specific context. However, it is important for optometrists to have pharmaceutical prescribing privileges because their professional roles are expected to expand and match these countries in accordance with the WCO Competency Framework [55], which aligns to the WHO Global Competency Framework [56].

While a cost–benefit analysis on evidence-based myopia interventions is unavailable, strong evidence from systematic reviews and meta-analyses supports the treatment approaches [29,37,38,39], given their cost-effectiveness in controlling the progression of myopia [30]. Where gaps in local data may exist, there are no detectable biases in the selected literature in this policy review and analysis. As evidence evolves, positions may shift according to current evidence. Further studies can be conducted to understand the utilisation of combined therapy across different socioeconomic groups.

## 5. Conclusions

This health policy review addresses the policy-level challenges to community-level interventions of myopia in Singapore. The policy analysis favours independent prescribing for optometrists as it is more likely to reduce the disease burden and generate cost savings to reduce the patients’ financial burdens. Optometrists are suitable for prescribing medications as they have the necessary knowledge, expertise, and training in using specialist diagnostic instruments. As Collaborative Prescribing has already been introduced to other healthcare professions in Singapore, this paper contributes to the existing literature by examining the current regulatory framework in view that a similar framework can be adapted for optometrists in the community to combat childhood myopia.

## Figures and Tables

**Table 1:** Policy Analysis of Independent Prescribing and Supplementary Prescribing in comparison with status quo using the Centers for Disease Control and Prevention (CDC) Policy Analysis Framework.

	Policy 1 Independent Prescribing	Policy 2 Supplementary Prescribing	Policy 3 Status Quo
Public Health Impact	(1) Increases access and reduces barriers by streamline treatment processes so that all tests can be completed within one visit; reduces wait time and inconvenience. (2) Children with myopia are at risk; high prevalence in Singapore (3) Likely to reduce health disparity due to lower treatment costs. (4) Evidence is strong concerning myopia control.	(1) Increases access and reduces barriers by streamline treatment processes so that all tests can be completed within one visit; reduces wait time and inconvenience. (2) Children with myopia are at risk; high prevalence in Singapore (3) Likely to reduce health disparity due to lower treatment costs. (4) Evidence is strong concerning myopia control.	(1) Delayed interventions due to barriers, (2) Children are not getting the most effective myopia control approaches. (3) Health disparity as a result of high treatment costs. (4) Strong evidence showing barriers to treatment.
Feasibility	Political (1) Pushback from ophthalmologists due to traditional mindset and vested interests. (2) Consumers may support lower cost treatment if it is made available (3) Patients may perceive service is poorer from optometrists (4) Substantial cost-savings to patient and substantially reduces healthcare costs.	Political (1) Ophthalmologists are more likely to agree with co-management due to shared fee structure. (2) Consumers may support as it may add an extra layer of safety in the prescribing. (3) Patients may be confused if tests need to be repeated. (4) Moderate cost-saving and has little impact on healthcare costs	Political (1) Ophthalmologists tend to favour status quo due to vested interests. (2) Consumers may worry that optometrists are not well trained to prescribe medications. (3) Patients may lack understanding on myopia control (4) Costs are high due to multiple separate visits to both optometrist and ophthalmologists
	Operational (1) Legislation and regulatory changes may be necessary (2) Two years to be enacted, implemented and enforced. (3) Uptake is likely and is scalable.	Operational (1) Legislation and regulatory changes may be necessary (2) Unpredictable due to contractual agreements and commercial interests. (3) Not likely to be sustainable due to possible contractual disagreements.	Operational (1) Legislation and regulatory changes not required (2) Not applicable. (3) Not likely to improve public access to treatment.
Economic and Budgetary Impact	Budget (1) Minimal costs required	Budget (1) Moderate costs required for system level changes	Budget (1) No impact
	Economic (1) Substantial cost-savings to patients (2) Potentially reduce healthcare cost and disease burden due to reduced prevalence of myopia and healthcare costs. (3) Good evidence showing that myopia control can work; data gap in some areas.	Economic (1) Moderate cost-savings to patients (2) Unlikely to reduce healthcare cost and disease burden (3) Good evidence showing that myopia control can work; data gap in some areas.	Economic (1) High treatment costs which is entirely out-of-pocket (2) Healthcare costs of myopia is high in Singapore (3) Evidence shows barriers are significantly hindering treatment uptake.

**Table 2:** Annual treatment cost comparison chart of combined therapy per patient under independent and supplementary prescribing by optometrists as compared with the status quo #.

Cost of Combined Therapy	Status Quo	Independent Prescribing	Supplementary Prescribing
Optical Shop + Public Hospital Pricing	S$3350	S$2800 (16% less)	S$3200 (5% less)
Optometry Clinic + Private Ophthalmology Clinic Pricing	S$4230	S$3180 (25% less)	S$3780 (10% less)

# The annual treatment costs are in Singapore dollars (S$). Fees will vary according to provider categories. For the purpose of comparison, the annual treatment costs of orthokeratology and atropine at status quo from optical shops (S$2500) were combined with those of public hospitals (S$850), respectively, and those optometry clinics (S$2680) were combined with those of private ophthalmology clinics (S$1550). Similarly, the annual treatment costs for independent prescribing and supplementary prescribing are calculated in the same manner based on the estimated average treatment costs presented in Table 3, under the assumption that the optometrist does not increase the charges during independent prescribing and that the ophthalmologist at public hospitals and private clinics would levy S$100 and S$150, respectively, due to fee-sharing during each visit under the co-management supplementary prescribing arrangement.

**Table 3:** Breakdown of estimated treatment and consultation costs per patient *.

	Atropine from Ophthalmologists		Orthokeratology from Optometrists	
	Private Clinic	Public Hospital	Optical Shop	Optometry Clinic
Consultation (First Visit)	S$300	S$170	Waived	S$180
Consultation (Subsequent)	S$250	S$130	Factored into treatment cost	
Treatment Cost	S$500	S$300	S$2500	S$2500
Annual Cost (Total)	S$1550	S$850	S$2500	S$2680

* The annual treatment and consultation costs in this table are in Singapore dollars (S$) and are estimated based on market research from the providers: optical shop, optometry clinic, private ophthalmology clinic and public hospital. The monthly treatment cost of atropine is approximately S$25 (after subsidies) and S$40 at public hospitals and private ophthalmology clinics, respectively. The annual cost of consultation is based on four quarterly visits. Consultation fees with optometrists at optical shops are typically waived or factored into the cost of treatments or charged nominally at optometry clinics, which are more specialised in offering myopia treatments.

## Appendix A

**Table A1:** Summary of the Benefits and Drawbacks of Prescribing Privileges for Optometrists.

Authors	Publication Year	Country (Geographical Region)	Treatment Domains	Benefits/Drawbacks
Jindal, Abdulrasid, Mulholland, et al. [64]	2024	United Kingdom (England, Scotland, Wales, and Northern Ireland)	Cataract, glaucoma, paediatrics, low vision, external, urgent care clinic, medical retina, and contact lenses.	Hospital optometrists with independent prescribing qualifications had a higher number of advanced skills compared to those without.
Carmichael, Abdi, Balaskas, Costanza, and Blandford [65]	2022	United Kingdom (England, Scotland, Wales, and Northern Ireland)	Glaucoma, cataract, and medial retina.	Additional training as part of independent prescribing for optometrists helps to reduce false-positive referrals and ease the strain at public hospitals.
Cottrell, North, Sheen, and Ryan [93]	2022	United Kingdom (Wales)	Glaucoma, anterior eye, dry eye, cataract, medical retina, and ocular motor balance.	Independent prescribing by optometrists during the 10-week COVID lockdown helped to reduce the burden on the hospital eye services.
Gunn, Creer, Bowen, et al. [67]	2022	United Kingdom (England, Scotland, Wales, and Northern Ireland)	Cornea, glaucoma, medical retina, cataract, diabetic eye disease, eye casualty, paediatrics, uveitis, neuro-ophthalmology, and laser surgery.	Hospital optometrists are often prescribing independently. A wide variety of clinical procedures or interventions are undertaken by hospital optometrists. A small number of hospital optometrists perform specific laser procedures, including selective laser trabeculoplasty.
MacIsaac, Naroo, and Rumney [94]	2022	United Kingdom (England)	Minor eye conditions include anterior eye, uvea, trauma, glaucoma, post-op inflammation, medical retina, and refractive errors.	With independent prescribing, more than 66% of patients from the hospital can be managed by optometrists in the community.
Ansari, Patel, and Harle [66]	2022	United Kingdom (England)	A variety of acute conditions from the emergency department	During the COVID lockdown, optometrists with independent prescribing privileges were able to safely and efficiently treat and manage the vast majority of urgent cases.
Jonuscheit, Geue, Laidlaw, et al. [85]	2021	United Kingdom (Scotland)	Antibacterials, anti-inflammatories and dry eye treatments.	Optometrists in the community are contributing to lessening the burden in primary care.
Spillane, Courtenay, Chater, et al. [72]	2021	United Kingdom (England, Scotland, Wales, and Northern Ireland)	Glaucoma, anterior eye, and medical retina	Optometrists with therapeutics training were more confident in diagnosing and managing specific ocular conditions. Trained and experienced independent prescriber optometrists are able to make appropriate clinical decisions. Poor remuneration, fear of litigation and time/cost of training were barriers.
El-Abiary, Loffler, Young, et al. [86]	2021	United Kingdom (Scotland)	Various conditions	With independent prescribing privileges, optometric referrals to public hospitals continued to rise. As age-related eye conditions become more prevalent, more patients require referral to public hospitals.
Todd, Bartlett, Thampy, et al. [73]	2020	United Kingdom (England)	General ophthalmology, emergency, uveitis cornea, surgical/vitreo-retina, glaucoma, medical retina, neuro-ophthalmology and oculoplastics.	Clinical decision-making by optometrists with independent prescribing privileges are concordant with ophthalmologists.
Steward, MacLure, and George [60]	2012	United Kingdom (England, Scotland, Wales, and Northern Ireland)	Not reported	Independent prescribing is safe and appropriate. Patient acceptability and satisfaction of independent prescribing was high.
Courtenay, Carey, and Stenner [61]	2012	United Kingdom (England, Scotland, Wales, and Northern Ireland)	Not reported	The low use of supplementary prescribing due to the greater co-working requirement with a medical doctor.

## Appendix B

**Table A2:** Quality of Evidence and Data from the Policy Review and Analysis on Pharmaceutical Prescribing Privileges for Optometrists Based on the Centers for Disease Control and Prevention (CDC) Policy Analysis Framework.

	Public Health Impact	Feasibility	Economic and Budgetary Impact	
			Budget	Economic
Policy 1 Independent Prescribing	High *	High *	More Favourable *	More Favourable *
Policy 2 Dependent Prescribing	Medium *	Medium *	Less Favourable *	Favourable *
Policy 3 Status Quo	Low *	Medium *	Favourable *	Favourable *

* No concerns about the amount or quality of data.
